# Stronger fertilization effects on aboveground versus belowground plant properties across nine U.S. grasslands

**DOI:** 10.1002/ecy.3891

**Published:** 2022-12-07

**Authors:** Adrienne B. Keller, Christopher A. Walter, Dana M. Blumenthal, Elizabeth T. Borer, Scott L. Collins, Lang C. DeLancey, Philip A. Fay, Kirsten S. Hofmockel, Johannes M. H. Knops, Andrew D. B. Leakey, Melanie A. Mayes, Eric W. Seabloom, Sarah E. Hobbie

**Affiliations:** ^1^ Department of Ecology, Evolution, and Behavior University of Minnesota Saint Paul Minnesota USA; ^2^ Department of Biology West Virginia University Morgantown West Virginia USA; ^3^ USDA‐ARS Rangeland Resources & Systems Research Unit Fort Collins Colorado USA; ^4^ Department of Biology University of New Mexico Albuquerque New Mexico USA; ^5^ USDA‐ARS Grassland Soil, and Water Research Laboratory Temple Texas USA; ^6^ Earth and Biological Sciences Directorate Pacific Northwest National Laboratory Richland Washington USA; ^7^ Department of Agronomy Iowa State University Ames Iowa USA; ^8^ Health & Environmental Sciences Department Xi'an Jiaotong‐Liverpool University Suzhou Jiangsu China; ^9^ Department of Plant Biology, Department of Crop Sciences University of Illinois at Urbana‐Champaign Urbana‐Champaign Illinois USA; ^10^ Department of Earth and Planetary Sciences University of Tennessee Knoxville Tennessee USA; ^11^ Environmental Sciences Division Oak Ridge National Laboratory Oak Ridge Tennessee USA

**Keywords:** belowground net primary productivity, nitrogen, Nutrient Network, nutrient pollution, phosphorus, root response

## Abstract

Increased nutrient inputs due to anthropogenic activity are expected to increase primary productivity across terrestrial ecosystems, but changes in allocation aboveground versus belowground with nutrient addition have different implications for soil carbon (C) storage. Thus, given that roots are major contributors to soil C storage, understanding belowground net primary productivity (BNPP) and biomass responses to changes in nutrient availability is essential to predicting carbon–climate feedbacks in the context of interacting global environmental changes. To address this knowledge gap, we tested whether a decade of nitrogen (N) and phosphorus (P) fertilization consistently influenced aboveground and belowground biomass and productivity at nine grassland sites spanning a wide range of climatic and edaphic conditions in the continental United States. Fertilization effects were strong aboveground, with both N and P addition stimulating aboveground biomass at nearly all sites (by 30% and 36%, respectively, on average). P addition consistently increased root production (by 15% on average), whereas other belowground responses to fertilization were more variable, ranging from positive to negative across sites. Site‐specific responses to P were not predicted by the measured covariates. Atmospheric N deposition mediated the effect of N fertilization on root biomass and turnover. Specifically, atmospheric N deposition was positively correlated with root turnover rates, and this relationship was amplified with N addition. Nitrogen addition increased root biomass at sites with low N deposition but decreased it at sites with high N deposition. Overall, these results suggest that the effects of nutrient supply on belowground plant properties are context dependent, particularly with regard to background N supply rates, demonstrating that site conditions must be considered when predicting how grassland ecosystems will respond to increased nutrient loading from anthropogenic activity.

## INTRODUCTION

A central question of ecology is what controls patterns of net primary productivity (NPP) and plant resource allocation, because NPP provides the energetic basis for nearly all life on Earth and is an important regulator of climate. Nitrogen (N) and phosphorus (P) commonly constrain terrestrial NPP (Elser et al., 2007; Harpole et al., 2011; LeBauer & Treseder, 2008; Vitousek et al., 2010). However, inputs of N and P to terrestrial ecosystems are increasing due to anthropogenic activities, such as agricultural fertilization and burning of fossil fuels, potentially altering nutrient limitation in some regions. Notably, N inputs are increasing at a much faster rate than are P inputs, resulting in shifts in ecosystem stoichiometry that are expected to have critical, yet still unclear, consequences for plant communities (Peñuelas et al., 2013). Although considerable work has demonstrated that aboveground net primary productivity (ANPP) and biomass tend to increase with increased N and P inputs (Elser et al., 2007; LeBauer & Treseder, 2008), much less is known about belowground plant responses to nutrient amendment, particularly root fluxes such as root productivity and turnover. Because roots are major contributors to soil carbon (C) storage, understanding belowground net primary productivity (BNPP) and biomass responses to changes in nutrient availability is essential to predicting carbon–climate feedbacks in the context of interacting global environmental changes.

Despite a relative paucity of data, recent global meta‐analyses show that belowground plant responses to nutrient amendment are highly variable. Belowground responses may vary in root biomass (absolute allocation belowground) or root mass fraction (relative allocation belowground), as well as rates of productivity and tissue turnover. Song et al. (2019) reported that both ANPP and root standing biomass, but not BNPP, increased with added N. In contrast, Peng et al. (2017) found that N addition did not significantly alter root standing biomass but suppressed root production and turnover rates. Although lower nutrient demand under fertilization may shift plants from being nutrient to light limited and thereby reduce relative plant allocation belowground, concurrent limitation by other resources (e.g., water, other nutrients) may intensify under fertilization and promote belowground allocation (Gleeson et al., 1992). In the absence of shifts in belowground allocation, overall growth should translate to increased belowground biomass. Additionally, both phenotypic plasticity and shifts in plant community composition may alter relative belowground allocation and rates of root production and turnover with changes in nutrient supply (Reynolds & D'Antonio, 1996). Thus, multiple and potentially counteracting factors make predictions of root responses to fertilization challenging.

Predicting plant responses to nutrient addition is of particular importance in grasslands, since they cover nearly a third of nonagricultural land and store a greater proportion of their plant biomass belowground compared to most other biomes (Gherardi & Sala, 2020; Poorter et al., 2012). Despite this, grasslands remain understudied compared to forests with respect to fertilization effects on roots, especially belowground productivity. For example, in meta‐analyses of N enrichment effects on root dynamics and root traits, <20% and 5% of the studies were in grasslands, respectively (Li et al., 2015; Peng et al., 2017). Additionally, most studies have focused solely on N, yet there is increasing evidence that colimitation by multiple nutrients is common across terrestrial ecosystems, including grasslands (Bracken et al., 2015; Du et al., 2020; Fay et al., 2015; Harpole et al., 2011). For example, Li et al. (2016) reported N + P addition enhanced both aboveground and belowground biomass more than did N addition alone across grasslands. In other cross‐site analyses, aboveground biomass was colimited by N and P at a majority of sites (Fay et al., 2015), whereas belowground biomass was limited by N but not by P or other nutrients (Cleland et al., 2019). However, it remains unknown whether belowground productivity—a key belowground flux that drives terrestrial carbon cycling and reflects how plants respond to their environment—is colimited by N and P, similarly to ANPP.

Moreover, meta‐analyses report considerable variation of fertilization effects across studies and, given most studies are carried out at a single site and methods often vary among studies, generalizable patterns of plant production and allocation responses to fertilization remain elusive. Such idiosyncratic results also likely reflect interactive effects of fertilization and site‐level factors such as climate, N deposition, and soil properties that have been shown to influence root properties (e.g., Hui & Jackson, 2006; Mccormack & Guo, 2014). However, which factors are important in explaining variation in grassland belowground response to fertilization cannot be untangled using meta‐analysis.

Belowground biomass contributes disproportionately to soil C in grasslands (Jobbágy & Jackson, 2000), and fertilization effects on root biomass may ultimately alter soil C storage (Fornara & Tilman, 2012). Improvements in soil C modeling have enabled explicit inclusion of root dynamics in many soil C models (e.g., CORPSE; Sulman et al., 2014), thereby improving our ability to predict soil C responses to global change. However, a lack of empirical data on root responses to nutrient supply currently hinders soil C modeling efforts. Thus, measures of root pool and flux responses to nutrient enrichment is critical to inform understanding of soil C dynamics (Keller et al., 2021).

The Nutrient Network (NutNet), an experiment replicating fertilization treatments across diverse grasslands, has greatly increased the potential to uncover generalities regarding how nutrient addition and site factors influence aboveground and belowground plant allocation patterns in grasslands. Recent work spanning 29 NutNet sites showed that root biomass responses to N addition varied as a function of light availability at the ground surface (Cleland et al., 2019); however, root productivity (a reflection of dynamic resource allocation in contrast to static root biomass) was not measured, leaving a gap in knowledge about rates of C flux belowground. Furthermore, Cleland et al. (2019) examined responses relatively early in the experiment, 3–5 years after fertilization began, providing important insights into early responses but no indication about longer‐term impacts. Yet, recent work in the NutNet experiment (Seabloom, Adler, et al., 2021) and a meta‐analysis (Liang et al., 2021) demonstrate that longer‐term (>8 years) biomass responses to N fertilization increase over time in grasslands. Thus, short‐term plant responses to fertilization may underestimate the effects of increased nutrient supply on grassland ecosystem functioning (e.g., soil C storage, nutrient and water cycling) over ecologically relevant time scales (Borer et al., 2017). Thus, examining longer‐term responses of both belowground biomass and productivity to nutrient supply across different grasslands will improve understanding of grassland ecosystem functioning under environmental change.

Here, we report on a study of plant belowground and aboveground responses to a decade of N and P fertilization across nine temperate U.S. grassland sites. We asked (1) how belowground plant properties, including root biomass and production, respond to N and P fertilization, (2) how environmental factors (e.g., climate, soil texture, atmospheric N deposition and soil C) mediate these belowground responses, and (3) whether belowground plant responses to N and P fertilization mirror those of aboveground plant responses. Overall, we hypothesized that nutrient addition would increase root production while also increasing root turnover due to reduced nutrient limitation. Across sites, we hypothesized that nutrient addition effects on plant C allocation patterns would be most pronounced at sites where water availability was most favorable for plant productivity and least pronounced in soils with high baseline fertility. Finally, we expected nutrient addition effects to be stronger aboveground compared to belowground.

## MATERIALS AND METHODS

### Research sites

**FIGURE 1:**
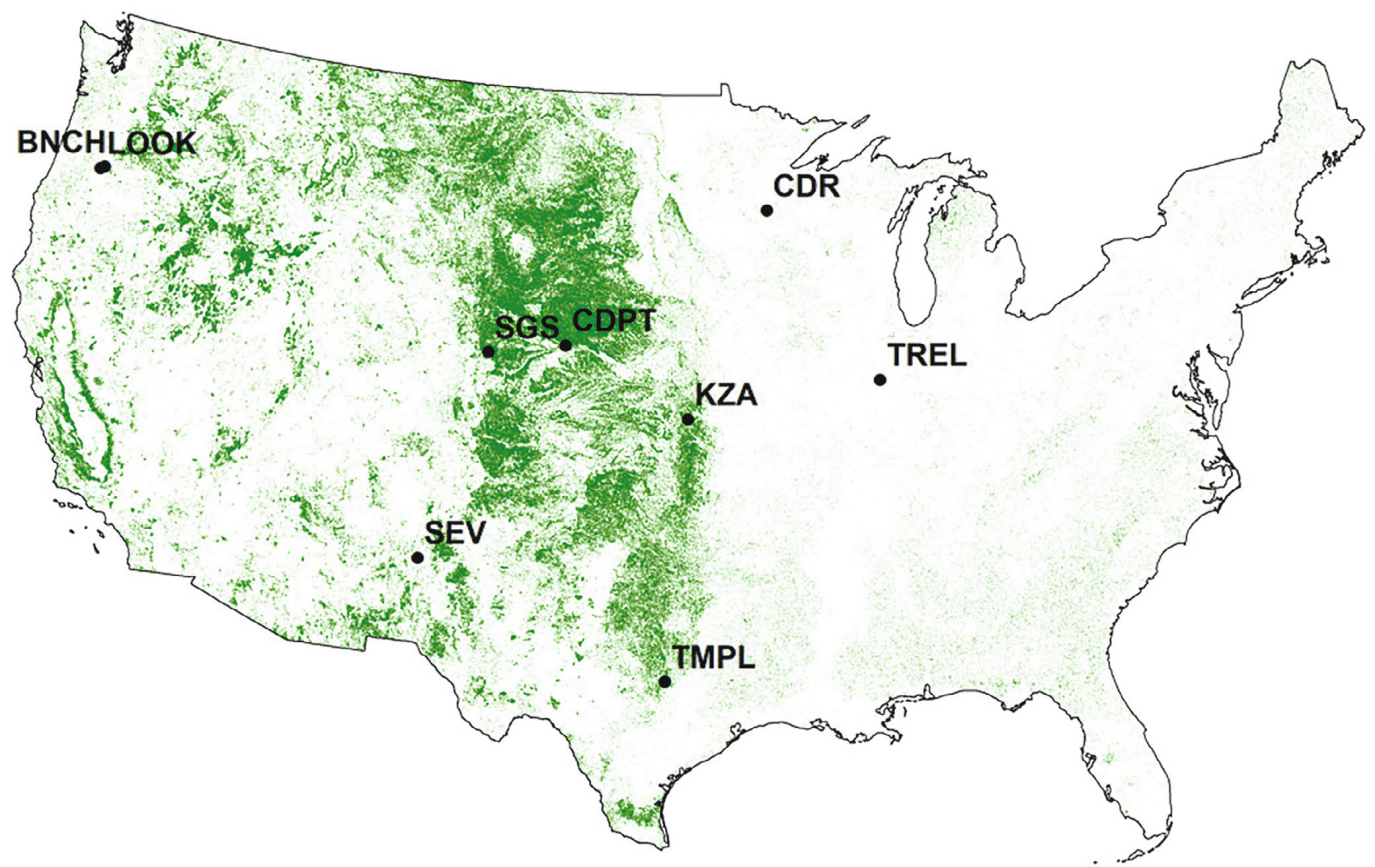
Map of continental United States with location of nine study sites overlaying a grassland/herbaceous land cover (green) from National Land Cover Database (2016). Site abbreviations as in Table 1.

The nine NutNet sites used in this study were chosen to span gradients of climate (e.g., mean annual precipitation: 252–1877 mm), soil properties (e.g., pH and texture), atmospheric N deposition (2.0–16.8 kg ha^−1^ year^−1^), and aboveground biomass (98–1173 g m^−2^), while also representing the widespread geographic distribution of North American grasslands (Table 1, Figure 1). Details of the network's experimental design are provided by Borer et al. (2014). Briefly, at each site, we sampled plots amended with N or P (alone and in combination), as well as control plots receiving no fertilizer. Treatments were replicated in three or four completely randomized blocks at each site. N and P were applied annually (10 g m^−2^ year^−1^) for 9–10 years prior to sampling. At most sites, N fertilizer was applied as ammonium nitrate (NH_4_NO_3_) in Treatment Year 1 and time‐released urea [(NH_2_)_2_CO] in subsequent years; exceptions included SEV and TMPL, which received ammonium nitrate in all years, and TREL, which received urea in all years. Previous work at six NutNet sites showed that the N source did not significantly affect aboveground biomass or richness (Seabloom et al., 2013). All sites received P fertilizer annually as triple super phosphate [Ca(H_2_PO_4_)_2_].

**TABLE 1:** Site properties for the nine sites included in this study.

Site and grassland type	Dominant plant species^a^	Latitude, longitude (°)	MAT (°C), MAP (mm)	Moisture index	Precipitation distribution^b^	Elevation (m)^c^	N deposition (kg N ha^−1^ year^−1^)^c^	Soil order	Soil texture (% sand, silt, clay)
Bunchgrass, OR (BNCH); montane grassland	*Carex pensylvanica*	44.28, −121.97	6.77, 1618	1.93	0.17	1318	2.84	Inceptisol	70.5, 26.6, 2.9
Cedar Creek LTER, MN (CDR); tallgrass prairie	*Poa pratensis, Andropogon gerardii, Elymus repens*	45.43, −93.21	6.34, 740	0.83	0.15	270	6.98	Entisol	88.9, 7.6, 3.5
Cedar Point Biological Station, NE (CDPT); shortgrass prairie	*C. filifolia, Bromus tectorum*	41.20, −101.63	9.64, 456	0.40	0.18	965	3.12	Mollisol	68.9, 21.6, 9.5
Konza LTER, KS (KZA); tallgrass prairie	*Schizachyrium scoparium, A. gerardii*	39.07, −96.58	11.6, 971	0.89	0.15	440	9.25	Mollisol	31.9, 49.8, 18.3
Lookout Ridge, OR (LOOK); montane grassland	*C. pensylvanica, Erigeron aliceae*	44.21, −122.13	6.9, 1877	2.31	0.17	1500	2.84	Andisol	69.0, 30.1, 0.9
Sevilleta LTER, NM (SEV); desert grassland	*Chrondrosum eriopodum, Salsola kali*	34.36, −106.69	13.1, 252	0.17	0.21	1600	1.96	Aridisol	83.6, 10.6, 5.8
Shortgrass Steppe LTER, CO (SGS); shortgrass prairie	*E. elymoides, C. duriuscula*	40.82, −104.77	8.95, 369	0.32	0.17	1650	3.12	Entisol	71.3, 15.1, 13.6
Temple, TX (TMPL); tallgrass prairie	*Sorghum halepense, Ambrosia trifida*	31.04, −97.35	19.4, 877	0.60	0.13	184	7.25	Mollisol	26.4, 34.3, 39.3
Trelease Prairie, IL (TREL); tallgrass prairie	*Solidago canadensis, Juncus* sp.	40.08, −88.83	11.1, 992	0.89	0.11	200	8.58	Mollisol	22.2, 62.2, 15.6

Abbreviation: LTER, Long Term Ecological Research Network.

^a^ Plant species with greater than 20% mean relative species abundance (mean fraction of species cover/total cover), calculated in 2016 for TMPL and 2017 for all other sites.

^b^ Precipitation distribution = precipitation in wettest month/MAP.

^c^ Atmospheric N deposition.

### Belowground and aboveground biomass and productivity

Belowground, we measured root standing biomass, root production, and root turnover at depths of 0–15 cm in each plot. Specifically, in fall 2016, three soil cores were extracted per plot (5 cm diameter, 15 cm depth). Soils were sieved to 2 mm, and then roots were removed using forceps, washed with deionized water, and weighed to 0.001 g after drying at 60°C for 48 h. Root standing biomass was calculated as the average root dry mass per plot on an areal basis. Root production was measured using root‐ingrowth cores. Immediately following soil coring, a rigid mesh polypropylene cylinder with the same dimensions as the soil core (Industrial Netting RN4465, Minneapolis, MN, USA) was placed in the soil core hole. The rigid mesh openings were 4.6 mm^2^ on the sides, with flexible and smaller‐holed window‐screen mesh sewn to the bottom of the cylinder to retain soil and allow for drainage (Industrial Netting XN3234, Minneapolis, MN, USA). Soil that was collected from cores in each plot was returned to the same plot following removal of roots and was mixed 2:1 with sand (to prevent compaction) before being placed in the ingrowth cores. The ingrowth cores were installed in fall 2016 and harvested in fall 2017. After harvesting, ingrowth core soil was sieved to 2 mm, and the roots were removed using forceps, washed with deionized water, dried at 60°C for 48 h, and weighed to 0.001 g. Net root production was calculated as root mass within each core at the time of harvesting and expressed on an areal basis. Root turnover was estimated as the ratio of root production to root standing biomass. Peak aboveground live biomass (hereafter, aboveground biomass) was sampled in two 0.1 × 1‐m strips in each plot. Peak biomass (a pool) is an imprecise estimate of ANPP (a flux), particularly in response to fertilization treatments, due to variable herbivory patterns across plots and sites, which can interact with fertilization effects. However, it is a useful approximation for comparing aboveground and belowground productivity.

### Abiotic and biotic covariates

The nine sites spanned the continental United States and varied widely in climate and edaphic factors, allowing us to test our hypotheses about interactions between site factors and nutrient enrichment effects. To capture how abiotic and biotic factors influenced plant responses to nutrient enrichment, we considered the predictors of aboveground and belowground plant biomass and production based on their importance in other work: soil pH and soil C stock at the plot level, soil % clay, atmospheric N deposition, moisture index (mean annual precipitation/potential evapotranspiration), mean annual precipitation (MAP), mean annual temperature (MAT), and precipitation seasonality at the site level. Soil pH, texture, and C stocks (0–15 cm) were measured by standard protocols, as described by Keller et al. (2021). Modeled atmospheric N deposition was determined for each site from Ackerman et al. (2019). For a time‐integrated metric of N deposition spanning the duration of this study, we averaged annual estimates of N deposition from 2014 to 2016.

A climate moisture index was calculated as the quotient of mean annual precipitation and potential evapotranspiration, spanning 1970–2000 (retrieved from CIGAR‐CSI/BIOCLIM database). Additional climate variables also were retrieved from the CIGAR‐CSI/BIOCLIM database, including MAT and MAP, precipitation in the driest and wettest months, precipitation variability (calculated as the coefficient of variation of precipitation across months), and precipitation seasonality (calculated as precipitation in the wettest month divided by mean annual precipitation).

### Data analysis

We were unable to recover belowground data for five plots, resulting in a final sample size of 115 plots across nine sites. We assessed fertilization effects on plant responses in three ways. First, to assess fertilization effects for each site, we calculated the log response ratio (log RR) for each plant property as the log ratio of the response variable in fertilized compared to control plots, e.g., log(+N/Control), for each block and then calculated mean log RR and standard error for each site. Nitrogen addition treatments included N only and +N+P plots, and P addition treatments included P only and +N+P plots. Response variables included aboveground peak biomass, root production, root biomass, root turnover, and root mass fraction (RMF; root biomass/total biomass).

Second, to assess fertilization treatment effects on plant properties while taking into account site variation, we constructed one linear mixed model for each plant response variable. For these models, N and P and their interaction were included as fixed effects, and block nested within site was treated as a random intercept. Weights were applied using the varIdent(form = ~1|site) function (lme function; nlme package) in models where this weighted variance structure improved the fit compared to the unweighted variance structure (based on visual inspection of residuals and ΔAIC > 3). This weighting structure allows different variances per site. Due to low sample size within sites, random slopes could not be included in the model structure. Prior to model fitting, each response variable was assessed for normality. Aboveground biomass and belowground biomass, productivity, and turnover were natural log transformed.

Third, to examine how climate and soil chemistry covariates in addition to fertilization individually influenced plant properties, we used the same linear mixed model approach as previously but also included one given covariate as an additional fixed effect with block nested within site as a random intercept. The initial model structure was as follows: Each response variable predicted by N, P, N + P, N + covariate, P + covariate, and N + P + covariate as fixed effects and block nested within site as random intercepts. Interactions between nutrient addition and covariates were assessed for statistical significance, and the interaction term was removed from the final model if α > 0.05. Covariates included N deposition, moisture index, precipitation seasonality, MAT, soil % clay, soil pH, and soil C. MAP was highly correlated with moisture index (*r* = 0.98) and therefore was not included. Owing to the high collinearity among precipitation seasonality metrics (i.e., precipitation in the driest and wettest months, precipitation variability, and precipitation seasonality), precipitation seasonality was selected as the single precipitation seasonality covariate for the regression analyses. Notably, precipitation seasonality was also highly negatively correlated with atmospheric N deposition (*r* = 0.80), which could reflect the inclusion of precipitation in the modeling of wet N deposition estimates (Vet et al., 2014). In general, high multicollinearity across covariates (as observed with Pearson correlation coefficients and high variance inflation factors) indicated that single covariate models were a better analysis tool for this data set rather than including multiple covariates in a single mixed model. In addition, the high number of covariates relative to the number of sites precluded including all covariates together in a single model. Because of pH data were missing from two plots, the sample size for these multivariate linear mixed models was 113 plots across nine sites. All data analysis was performed in R version 3.6.1 (R Foundation for Statistical Computing, 2013).

## RESULTS

**FIGURE 2:**
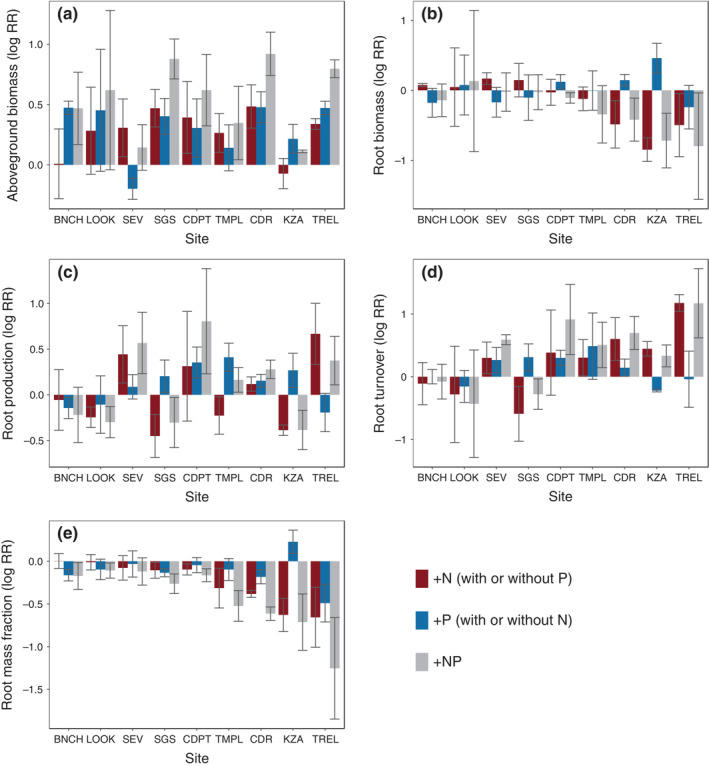
Log response ratio (log RR) of plant properties to N addition (red bars), P addition (blue bars), and N and P addition together (gray bars) for each site. Bars represent mean log RR for each site, with standard error bars shown in dark gray. Sites are ordered by increasing atmospheric N deposition.

**FIGURE 3:**
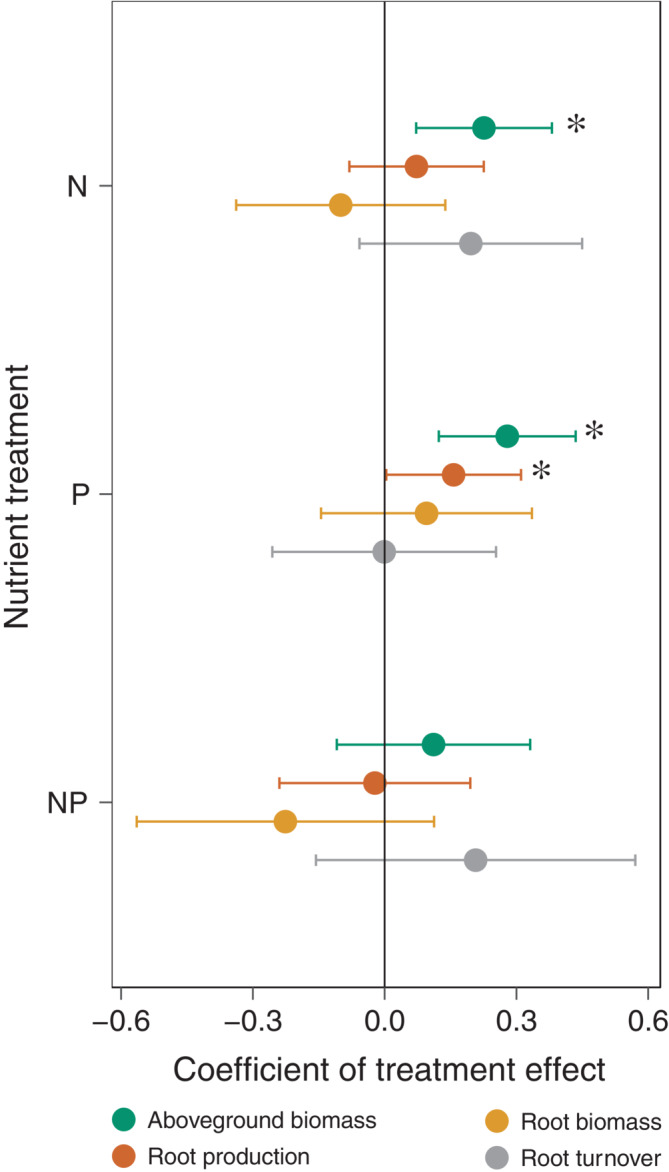
Mean parameter estimates and 95% confidence intervals for nutrient treatment effects in linear mixed models predicting aboveground biomass (green), root standing biomass (brown), root production (yellow), and root turnover (gray) where nutrient treatments were fixed effects and blocks nested within sites were random intercepts. * indicates parameter estimates that were statistically different from 0 with alpha = 0.05 (i.e., the 95% confidence intervals do not cross 0). The N × P interaction (NP) is compared to the sum of the effect of N and P alone, with zero indicating additivity.

We examined how N or P amendment (alone and in combination) affected aboveground and belowground plant pools and fluxes across nine NutNet grassland sites spanning the continental United States. Overall, aboveground biomass responded stronger to fertilization than did belowground plant properties, with the addition of N or P alone stimulating aboveground biomass across sites by 30% and 36%, respectively. Phosphorus addition increased root production by 15% across sites. In contrast to our expectations, we detected no overall effect of N or P fertilization on root biomass or root turnover (with or without site as a random effect, but without covariates). Aboveground and belowground responses to fertilization were not correlated, and root biomass and root production were uncorrelated with one another (*p* > 0.10) (Figures 2 and 3; Appendix S1: Table S1, Figure S1).

**FIGURE 4:**
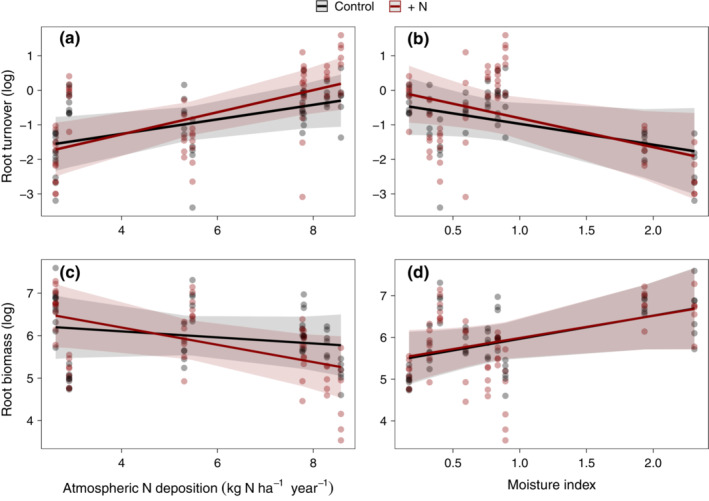
Interactive effects of +N and atmospheric N deposition on (a) root turnover (root biomass/root productivity) and (c) root biomass and +N and moisture index (MAP/PET) on (b) root turnover and (d) root biomass. Regression lines show predicted conditional effects from linear mixed models of +N and N deposition on plant properties. Shaded regions show 95% confidence intervals.

When covariate effects were included in statistical models, N and P increased aboveground biomass, with the strength of P effects depending on both climate (precipitation seasonality, moisture index, MAT) and soil pH (Table 2). Specifically, P effects on aboveground biomass tended to be greatest at cool, moist sites with an even distribution of rainfall and acidic soils, although these interactions of site conditions with elevated P effects were weak. Belowground, N and P supply emerged as significant predictors of root turnover and root production, respectively. There were weak but significant interactive effects of N and both N deposition or precipitation seasonality on root turnover (Figure 4a). Specifically, N effects on root turnover were highest at sites with high N deposition or low intra‐annual variability in precipitation (i.e., low precipitation seasonality). The covariate model including N deposition explained more than twice as much variation in the fixed effects as did the model including precipitation seasonality (N deposition: *R*_m_^2^ = 0.41, *R*_c_^2^ = 0.88; precipitation seasonality: *R*_m_^2^ = 0.17, *R*_c_^2^ = 0.86). Similarly, the interaction between N addition and moisture index was only marginally significant and was less effective for predicting root turnover than N deposition (moisture index: *R*_m_^2^ = 0.26, *R*_c_^2^ = 0.87; Figure 4b). Interactive effects of nutrient supply and covariates on root production were negligible. The effect of N supply on root biomass was mediated by N deposition, precipitation seasonality, and plant diversity separately, as observed from single‐covariate models. N deposition exhibited the strongest interactive effect of these three covariates, with added N decreasing root biomass at sites with high N deposition (Figure 4c). Moisture index did not significantly affect root biomass or interact with the treatments (Figure 4d).

**TABLE 2:** Results of nutrient treatment‐covariate statistical mixed‐effects full‐factorial N × P models predicting effects of fertilization on belowground plant properties.

Factor	AG biomass (ln)		Root production (ln)		Root biomass (ln)		Root turnover (ln)	
	*F*	*p*	*F*	*p*	*F*	*p*	*F*	*p*
N deposition^a^								
N dep	4.22	0.08	5.51	0.05	1.97	0.20	7.20	**0.03**
N:N dep	NA	NA	NA	NA	19.68	***<0.001***	11.69	***<0.001***
N	27.23	***<0.001***	1.71	0.19	3.47	0.07	16.27	***<0.001***
P	39.70	***<0.001***	7.16	***0.01***	0.44	0.51	0.55	0.46
N:P	0.68	0.41	0.11	0.75	2.60	0.11	0.96	0.33
Precip dist^b^								
Precip dist	3.17	0.12	1.15	0.32	0.68	0.44	1.61	0.24
N:Precip dist	NA	NA	NA	NA	11.53	<0.001	6.39	***0.01***
P:Precip dist	4.40	***0.04***	NA	NA	NA	NA	NA	NA
N	28.01	***<0.001***	1.72	0.19	1.91	0.17	18.17	***<0.001***
P	36.41	***<0.001***	7.12	***0.01***	0.74	0.39	0.46	0.50
N:P	0.48	0.49	0.10	0.75	2.45	0.12	0.71	0.40
Moisture^c^								
Moisture	0.01	0.92	0.57	0.48	3.05	0.12	3.34	0.11
P:Moisture	8.60	***0.00***	NA	NA	NA	NA	NA	NA
N	26.69	***<0.001***	1.73	0.19	1.20	0.28	12.29	***<0.001***
P	40.52	***<0.001***	7.11	***0.01***	0.82	0.37	1.19	0.28
N:P	0.79	0.38	0.10	0.75	2.27	0.14	0.81	0.37
MAT^d^								
MAT	2.49	0.16	0.01	0.91	2.18	0.18	1.02	0.35
P:MAT	6.86	***0.01***	NA	NA	NA	NA	NA	NA
N	27.44	***<0.001***	1.73	0.19	1.21	0.27	12.36	***<0.001***
P	40.87	***<0.001***	7.11	***0.01***	0.81	0.37	1.19	0.28
N:P	0.80	0.37	0.10	0.75	2.27	0.14	0.81	0.37
pH^e^								
pH	0.19	0.66	1.56	0.22	3.45	0.07	0.90	0.35
P:pH	10.12	***<0.001***	NA	NA	NA	NA	4.23	***0.04***
N	30.07	***<0.001***	1.94	0.17	1.43	0.24	14.86	***<0.001***
P	40.38	***<0.001***	7.73	***0.01***	0.80	0.37	0.93	0.34
N:P	0.73	0.39	0.09	0.76	2.29	0.13	0.64	0.43
% clay^f^								
% clay	2.96	0.09	0.01	0.92	0.94	0.34	0.15	0.70
N:% clay	NA	NA	4.94	***0.03***	NA	NA	NA	NA
N	27.33	***<0.001***	1.38	0.24	1.23	0.27	12.43	***<0.001***
P	39.76	***<0.001***	8.20	***0.01***	0.80	0.37	1.15	0.29
N:P	0.84	0.36	0.13	0.72	2.34	0.13	0.83	0.37
Soil C stock^g^								
Soil C stock	4.63	**0.03**	1.64	0.20	3.05	0.08	5.36	**0.02**
N	26.81	***<0.001***	1.28	0.26	1.32	0.25	11.62	***<0.001***
P	42.69	***<0.001***	6.38	***0.01***	0.77	0.38	1.30	0.26
N:P	0.55	0.46	0.02	0.89	2.43	0.12	1.80	0.18

*Note*: Models were initially fit with all interactions between individual covariates and nutrient treatments, but interaction terms were removed from final models when not significant (α > 0.05); the interactions that were removed are indicated in the table as “NA.” Terms that are statistically significant are shown in bold (α < 0.05; covariates alone: bold without italics, nutrient treatments alone, or interacting with covariates: bold with italics). The marginal and conditional *R* ^2^ values (*R*_m_^2^ and *R*_c_^2^, respectively) for each model are shown in the footnotes. Abbreviated names of factors include aboveground biomass (AG biomass), atmospheric N deposition (N dep), precipitation distribution (Precip dist), and moisture index (moisture).

^a^ AG biomass *R*_m_^2^ = 0.32, *R*_c_^2^ = 0.85; root production *R*_m_^2^ = 0.23, *R*_c_^2^ = 0.55; root biomass *R*_m_^2^ = 0.21, *R*_c_^2^ = 0.88; root turnover *R*_m_^2^ = 0.42, *R*_c_^2^ = 0.86.

^b^ AG biomass *R*_m_^2^ = 0.27, *R*_c_^2^ = 0.83; root production *R*_m_^2^ = 0.086, *R*_c_^2^ = 0.57; root biomass *R*_m_^2^ = 0.11, *R*_c_^2^ = 0.88; root turnover *R*_m_^2^ = 0.18, *R*_c_^2^ = 0.86.

^c^ AG biomass *R*_m_^2^ = 0.084, *R*_c_^2^ = 0.90; root production *R*_m_^2^ = 0.046, *R*_c_^2^ = 0.57; root biomass *R*_m_^2^ = 0.23, *R*_c_^2^ = 0.87; root turnover *R*_m_^2^ = 0.25, *R*_c_^2^ = 0.83.

^d^ AG biomass *R*_m_^2^ = 0.25, *R*_c_^2^ = 0.88; root production *R*_m_^2^ = 0.014, *R*_c_^2^ = 0.58; root biomass *R*_m_^2^ = 0.19, *R*_c_^2^ = 0.88; root turnover *R*_m_^2^ = 0.11, *R*_c_^2^ = 0.84.

^e^ AG biomass *R*_m_^2^ = 0.08, *R*_c_^2^ = 0.88; root production *R*_m_^2^ = 0.036, *R*_c_^2^ = 0.58; root biomass *R*_m_^2^ = 0.067, *R*_c_^2^ = 0.87; root turnover *R*_m_^2^ = 0.054, *R*_c_^2^ = 0.80.

^f^ AG biomass *R*_m_^2^ = 0.25, *R*_c_^2^ = 0.85; root production *R*_m_^2^ = 0.028, *R*_c_^2^ = 0.58; root biomass *R*_m_^2^ = 0.086, *R*_c_^2^ = 0.87; root turnover *R*_m_^2^ = 0.041, *R*_c_^2^ = 0.83.

^g^ AG biomass *R*_m_^2^ = 0.13, *R*_c_^2^ = 0.88; root production *R*_m_^2^ = 0.042, *R*_c_^2^ = 0.54; root biomass *R*_m_^2^ = 0.10, *R*_c_^2^ = 0.85; root turnover *R*_m_^2^ = 0.18, *R*_c_^2^ = 0.83.

Root properties varied substantially across sites in the unfertilized plots (Appendix S1: Figure S1, Table S2). Root production varied more than fourfold across sites (74–416 g m^−2^ year^−1^) and root standing biomass varied sixfold (150–932 g m^−2^). Root mass fraction (root biomass/total biomass) varied fourfold from 0.18 to 0.76. The measured abiotic and biotic factors largely did not explain this cross‐site variability, as evidenced by the small proportion of total variation explained by the fixed effects (i.e., *R*_m_^2^) in the multivariate linear mixed models (Table 2).

## DISCUSSION

### Nutrient effects on plant properties

We examined the effects of long‐term (ca. 10 years) N and P fertilization (alone and in combination) on aboveground and belowground plant pools and fluxes across nine NutNet grassland sites spanning a wide range of climatic and edaphic conditions. Overall, fertilization effects were more consistent and pronounced on aboveground biomass compared to belowground plant biomass and production (0–15 cm depth). Although the interactive effects of fertilization and site factors varied depending on the pool or flux of interest, N addition influenced root biomass and turnover, P addition increased root production, and both N and P stimulated aboveground biomass.

P addition stimulated root production, and although this effect varied by site, it was not mediated by any measured site factors. This result that P—but not N—fertilization stimulated production partially supported our prediction that increased nutrient supply would promote greater root production. The lack of a root production response to N is consistent with both a previous single‐site study at one of our focal sites in the desert southwestern United States (SEV) (Ladwig et al., 2012) and a broader meta‐analysis where N addition did not affect root production (Peng et al., 2017). However, plant responses to long‐term increases in P supply can reflect the plastic responses of C allocation or changes in community‐level plant traits because of shifts in plant community composition. For example, most grassland plant species associate with arbuscular mycorrhizal fungi (AMF) to enhance P uptake, and these species may reduce fungal colonization in favor of root proliferation under increased P supply (Smith & Smith, 2011), although recent work across NutNet sites shows little effect of P supply on AMF diversity and abundance (Kasanke et al., in review; Lekberg et al., 2021). Regardless of AMF colonization rates and in accordance of the growth rate hypothesis, elevated P availability may allow for greater plant tissue growth given that ribosomes (the site of protein synthesis) are rich in P (Elser et al., 1996). However, there is currently limited empirical support for the growth rate hypothesis in plants (Lambers, 2022; Matzek & Vitousek, 2009). Although fertilization effects on plant community composition were beyond the scope of this study, shifts in plant community composition could also alter plot‐level root production due to differences in species‐specific root traits. Indeed, belowground traits related to root production and nutrient foraging strategies have been shown to vary widely across plant functional groups and individual species (e.g., Kembel & Cahill, 2011; Levang‐Brilz & Biondini, 2003). Given  evidence of colimitation in multiple studies of grassland nutrient limitation (e.g., Craine et al., 2008; Fay et al., 2015), root responses to P fertilization may also be partially driven by increased calcium availability, as P was applied as Ca(H_2_PO_4_)_2_. Overall, we showed a general trend of elevated P supply stimulating root production across different grassland habitats, yet the mechanism(s) driving this pattern requires further research.

### Interactions between fertilization and site factors

Although P stimulated root production across sites independent of local environmental conditions, root responses to N fertilization depended on site conditions. Contrary to our hypothesis, the strength of nutrient effects on root biomass was not mediated by climate or soil C, as indicated by the low marginal *R* ^2^ values in the linear mixed‐effects models that included these factors as fixed effects. Instead, atmospheric N deposition emerged as the most important site factor determining N effects on root biomass and turnover. However, given the strong correlation between precipitation and N deposition, the importance of precipitation in driving root patterns warrants further exploration.

We found a negative relationship between atmospheric N deposition and root biomass responses to N addition, such that fertilization tended to increase root biomass at sites exposed to low rates of N deposition but decrease root biomass at sites with high N deposition. The interactive effects of N addition and N deposition on root biomass resulted from a strong positive relationship between N deposition and root turnover, with N addition further increasing root turnover at sites with high N deposition, combined with no effect of N addition on root production. This pattern supports the conceptual model of tissue lifetime efficiency, which predicts that root lifespan should decrease under plant N saturation due to the higher relative C cost of tissue maintenance compared to root N uptake (Smithwick et al., 2013). Although N deposition was highly correlated with precipitation patterns, and this pattern may be partially driven by root responses to water rather than N, N deposition was a better predictor of root responses to N than were precipitation variables. The effect of N deposition could indicate a shift toward N saturation at the high N deposition sites (Aber et al., 1998; Lovett & Goodale, 2011; Peng et al., 2020), although total soil N did not increase with N addition in this study (Keller et al., 2021). However, future soil N saturation could have important ramifications for predicting the long‐term effects of nutrient loading on grassland communities belowground. If atmospheric N deposition remains unabated at sites with high N input rates, plant C allocation belowground might decrease (Hendricks et al., 1993). This could consequently decrease C supply to microbes, suppressing microbial growth and turnover and reducing microbial contributions to soil organic matter pools (Deng & Liang, 2022). Thus, weakened plant–microbe interactions in N‐saturated soils could have downstream effects that reduce soil C allocation and storage. Taken together, our results suggest that N addition may alter short‐term root dynamics in grasslands, with the nature of the effect dependent on site‐specific N deposition rates.

The wide among‐site variation in response to identical fertilization treatments in this study suggests that the differences in fertilization response in belowground plant properties in this and other studies (Li et al., 2015; Peng et al., 2017) are likely due to site‐level constraints on these responses. Regarding this point, plant properties varied widely across sites regardless of nutrient treatment, with measured climate and soil factors explaining relatively little of this cross‐site variation. Unmeasured site differences in the dominant initial plant functional type (e.g., grasses with C3 vs. C4 photosynthetic pathways) or species and shifts in plant functional types or species due to nutrient amendment could have influenced site‐specific belowground patterns. Our focus on shallow (0–15 cm) roots may have exaggerated such site differences due, in part, to site differences in rooting depth. The proportion of total roots found in the top 15 cm undoubtedly varies across sites (Schenk & Jackson, 2002), and fertilization may shift the relative proportion of shallow roots. Consequently, the total fraction of root biomass captured by sampling to 15 cm depth (i.e., the depth sampling bias) may vary across both treatments and sites. Nonetheless, our standardized treatments and sampling across a wide range of site conditions, along with results from meta‐analyses (Li et al., 2015; Peng et al., 2017), suggest that weak and site‐specific belowground responses to nutrient addition are characteristic across grasslands.

### Belowground versus aboveground plant responses to fertilization

In accordance with a growing body of work, belowground plant responses to fertilization were weaker than aboveground biomass responses. In a temperate grassland in China, Wang et al. (2019) found that total NPP responses to N addition were driven primarily by aboveground rather than belowground plant responses. Similarly, in a cross‐biome meta‐analysis, Li et al. (2016) showed that fertilization of either N or P stimulated aboveground biomass more than belowground biomass. On the one hand, weaker belowground compared to aboveground responses to N and P supply may reflect plant reliance on roots, not just for uptake of these nutrients but also of water and other essential elements, as well as roots' role in storage and structural support. Therefore, if other belowground resources are limiting, plants may not significantly alter C allocation to roots under changes in N and P supply. Moreover, total plant C allocation belowground includes C supply to mycorrhizal fungi and rhizodeposition in addition to root biomass (Kuzyakov & Domanski, 2000), which may be responsive to nutrient addition in ways that are not revealed through biomass measures. Finally, accurate measurement of plant biomass and production is challenging belowground. Because of the need to minimize plot disturbance, our belowground estimates do not represent as much ground area as that sampled for aboveground biomass, do not account for deep roots, very fine roots, and root hairs that are difficult to separate from soil but are important for plant resource uptake, and do not consider phenological patterns that may differ from aboveground phenology (Finzi & Abramoff, 2015). Thus, improved methodologies for accurately quantifying belowground C allocation are needed to fully understand how nutrient supply drives ecosystem C cycling. However, given increasing evidence of the importance of root contributions to slow‐cycling soil C pools (Rasse et al., 2005; Sokol & Bradford, 2019) along with recent work showing minimal effects of nutrient supply on soil C stocks in these grasslands (Keller et al., 2021), the weaker belowground compared to aboveground plant responses observed in this study warn that enhanced aboveground productivity under fertilization may not stimulate soil C sequestration in natural grasslands.

### Outstanding questions

Further work exploring responses of belowground properties to gradients in nutrient supply will be informative, including studies of how responses vary with fertilization rates, over longer time scales (i.e., many decades), and sampling to greater depth. In this study, a single high level of fertilization was applied annually for ca. 10 years. The dose and frequency of nutrient addition may influence both the direction and magnitude of plant responses, although existing research on this point is inconclusive. For example, Wang et al. (2019) found that belowground plant responses were greater under low compared to high loads of N addition, whereas Li et al. (2016) reported the opposite effect. Regardless of dose, root responses to fertilization can also vary interannually and with time since initial fertilization (e.g., Adair et al., 2009; Seabloom, Borer, et al., 2021). Although our treatments represent a decade of altered nutrient supply, our estimates of root pools and fluxes were limited to a single year, and it is unclear how consistent the treatment effects we report here might be over time. However, the spatial variability of root properties is commonly much greater than temporal variability (Hui & Jackson, 2006), suggesting that the cross‐site patterns observed here likely persist across years. Our measures of root properties were limited to surface soils (0–15 cm), and distinct root patterns could emerge at depth (Jackson et al., 1996; Mueller et al., 2013). There could be important interactions between climate, fertilization responses, and root responses, as wetter sites have been shown to store a greater proportion of root biomass in surface soils (Schenk & Jackson, 2002), and nutrient supply could alter root depth profiles differently in differing climates. Nevertheless, prior work at many of our sites showed that the majority of root biomass was in surface soils (see SI table 2 in Cleland et al., 2019), and root responses to fertilization are expected to be greatest in surface soils where plant nutrient foraging is typically greatest. Finally, further exploration of the distinct belowground responses to changes in N versus P supply observed in this study will improve predictive understanding of root responses to environmental change.

## CONCLUSION

Overall, we found differential effects of fertilization on aboveground compared to belowground plant properties. Both N and P addition stimulated aboveground biomass, and P addition also stimulated root production. The effect of N supply on root properties depended on site conditions. Atmospheric N deposition emerged as the strongest predictor of root properties among sites, and site‐level N deposition interacted with additional N supply to determine among‐site variation in root biomass and turnover. Inclusion of atmospheric N deposition may improve predictive models of grassland ecosystem C cycling.

## CONFLICT OF INTEREST

The authors declare no conflict of interest.

## Supporting information


Appendix S1

## Data Availability

Data (Hobbie, 2021) are available from the Environmental Data Initiative at https://doi.org/10.6073/pasta/7f984c2ed9e63754577ee711e6d74a6e.
