# Supplementary material for: Stronger fertilization effects on aboveground versus belowground plant properties across nine U.S. grasslands
Source: Ecology. 2022 Dec 7;104(2):e3891. doi: 10.1002/ecy.3891 (PMC10078332; doi:10.1002/ecy.3891)
Supplement: Supplementary file 1 — Appendix S1 [file ECY-104-0-s001.pdf]

Supporting Information

Journal Name: Ecology

Article Title: Stronger fertilization effects on aboveground versus belowground plant properties across nine U.S. grasslands

Authors:

Keller, Adrienne B., Christopher A. Walter, Dana M. Blumenthal, Elizabeth T. Borer, Scott L. Collins, Lang C. DeLancey, Philip A. Fay, Kirsten S. Hofmockel, Johannes M H Knops, Andrew D.B. Leakey, Melanie A. Mayes, Eric W. Seabloom, Sarah E. Hobbie.

Appendix S1

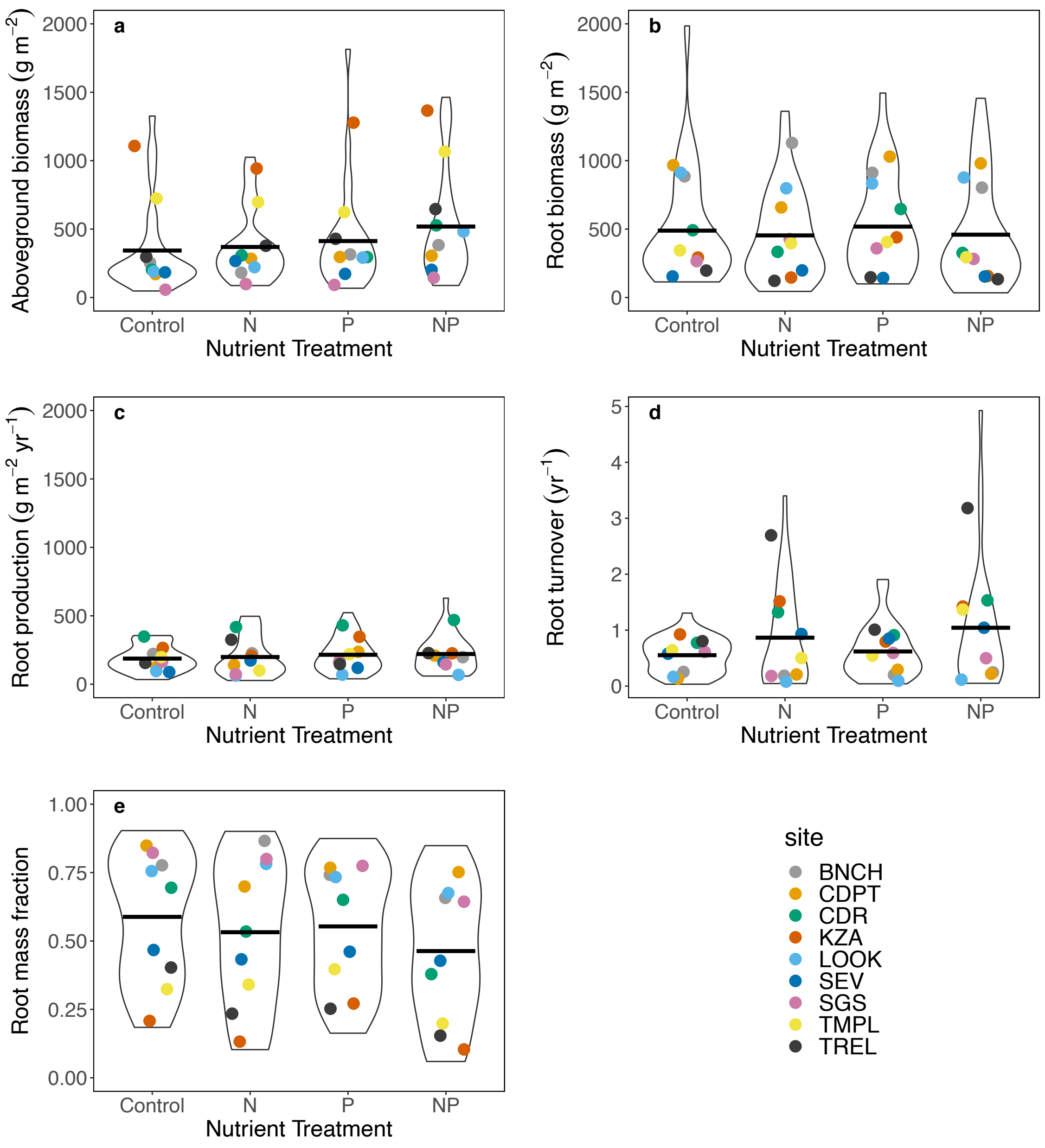

Figure S1. Effects of nutrient addition on aboveground and belowground plant properties. a) aboveground peak biomass, b) root standing biomass, c) root production, d) root turnover, and e) root mass fraction (root biomass/total biomass). Violin plots show the shape of the distribution across sites of plant responses to a given nutrient treatment, with the mean treatment response shown by a black bar. Individual colored points indicate site means for a given treatment.

Table S1. Treatment means (± SE) of plant properties across nine Nutrient Network sites.

Treatment	ANPP (g m^{-2} yr^{-1})	Root production (g m-2 yr-1)	Root biomass (g m^{-2})	Root turnover (yr^{-1})	Root mass fraction	Root productivity fraction
Control	343 (±61.9)	187 (±17.7)	489 (±76.3)	0.552 (±0.0590)	0.588 (±0.0429)	0.413 (±0.0343)
+ N	369 (±48.3)	199 (±27.3)	454 (±62.2)	0.866 (±0.166)	0.532 (±0.0482)	0.363 (±0.0331)
+ P	413 (±69.8)	215 (±24.4)	518 (±68.7)	0.619 (±0.0900)	0.553 (±0.0417)	0.383 (±0.0330)
+ NP	519 (±74.1)	221 (±25.2)	459 (±73.3)	1.04 (±0.212)	0.463 (±0.0459)	0.338 (±0.0294)

Table S2. Site means (± SE) of plant properties in unfertilized plots across nine Nutrient Network sites.

Site	Aboveground biomass (g m^{-2})	Root production (g m-2 yr-1)	Root biomass (g m^{-2})	Root turnover (yr^{-1})	Root mass fraction
BNCH (Oregon)	254 (±65.8)	221 (±12.7)	855 (±114)	0.254 (±0.0176)	0.776 (±0.0527)
CDR (Minnesota)	203 (±10.8)	348 (±3.82)	492 (±95.0)	0.773 (±0.112)	0.694 (±0.0350)
CDPT (Nebraska)	171 (±31.0)	127 (±47.2)	967 (±74.5)	0.135 (±0.0512)	0.849 (±0.0293)
KZA (Kansas)	1108 (±130.)	265 (±52.1)	292 (±44.5)	0.924 (±0.194)	0.208 (±0.0119)
LOOK (Oregon)	190 (±22.5)	95.9 (±21.0)	912 (±538)	0.167 (±0.0638)	0.756 (±0.0726)
SEV (New Mexico)	184 (±43.1)	88.9 (±12.5)	154 (±20.1)	0.578 (±0.0437)	0.467 (±0.0257)
SGS (Colorado)	57.1 (±4.77)	164 (±30.2)	266 (±16.8)	0.611 (0.0842)	0.822 (±0.0182)
TMPL (Texas)	724 (±146)	198 (±34.5)	344 (±65.5)	0.643 (±0.189)	0.324 (±0.0114)
TREL (Illinois)	297 (±55.0)	155 (±19.3)	197 (±24.6)	0.804 (±0.0956)	0.403 (±0.0382)
